# Do unequal societies cause death among the elderly? A study of the health effects of inequality in Swedish municipalities in 2006

**DOI:** 10.3402/gha.v6i0.19116

**Published:** 2013-01-16

**Authors:** Sören Edvinsson, Erling Häggström Lundevaller, Gunnar Malmberg

**Affiliations:** 1 Centre for Population Studies, Umeå University, Umeå, Sweden; 2 Ageing and Living Conditions Programme, Umeå University, Umeå, Sweden; 3 Department of Statistics, Umeå University, Umeå, Sweden; 4 Department of Geography and Economic History, Umeå University, Umeå, Sweden

**Keywords:** income inequality, mortality, Gini index, Sweden, municipality level, old age

## Abstract

**Background:**

A lively public and academic debate has highlighted the potential health risks of living in regions characterized by inequality. Research provides an ambiguous picture, however, with positive association between income equality and health mainly being found on higher levels of geographical division, such as nations, but rarely at local level.

**Methods:**

We examined the association between income inequality (using the Gini coefficient) and all-cause mortality in Swedish municipalities in the 65–74 age group. A multi-level analysis was applied and controlled for by variables including individual income and average income level in the municipality. The analyses were based on individual register data on all residents born between 1932 and 1941, outcomes were measured for the year 2006.

**Results:**

Lower individual income and lower average income in the municipality of residence were associated with significantly increased mortality. We found an association between income inequality and mortality with excessive deaths in unequal municipalities after controlling for mean income level and personal income. The results from the analysis of individual data differed substantially from the analysis of aggregate data.

**Conclusions:**

Income inequality was significantly associated with mortality in the age group 65–74 at municipality level. The association is small compared to many other variables, but is not negligible. Even in a comparatively equal society like Sweden, the potential effects of income inequality on mortality at the local level warrant consideration.

A recurring topic in contemporary social science and public health literature is the impact of socioeconomic inequality on health outcomes (1–3). Though many transitional economies foresee improved public health as their economies grow, a widening social gap may partly counter these trends. Previous research has shown how poverty and poor living conditions hinder the possibilities for good health in low-, middle-, and high-income countries, although the mechanisms and consequences may differ by geographical context and socioeconomic conditions (4).

With the almost universal trend of population ageing, a growing social gap among the elderly has become a key challenge for global health. On the one hand, living longer is a sign of health, but, on the other, it may result in longer periods of ill-health later in life, with increasing costs and suffering for individuals and society. In high-income countries, health problems evidently appear more often in old age, and longer lives may result in a process of cumulative advantages and disadvantages (5). A consequence of an ageing population may well be a widening social gap in terms of health outcomes and living conditions among old people. However, the determinants of the social health gradient among the elderly have also become topical issues in transitional economies experiencing epidemiological transition and population ageing.

Of the many factors influencing health outcomes in old age, conditions related to environment are depicted as essential (6), particularly in low-income countries (4). Nevertheless, poor socioeconomic environments and segregation have also been identified as potential determinants of health outcomes in low-, middle-, and high-income countries, and World Health Organization has recognized this theme as one of the most important challenges for future health work (4). More recently, the question of inequality itself has been brought to the fore in the academic debate. Wilkinson and Pickett have claimed that living in areas characterized by income inequality, in itself, has a negative impact on health outcomes (7, 8). Not only do poverty and living in a poor socioeconomic environment result in poor health; living in a country, region, or neighborhood characterized by inequality would, in line with these arguments, add to the health problems. If income inequality in itself triggers health problems in developed societies, this is indeed also a concern for countries now experiencing an epidemiological transition.

The crucial importance of socioeconomic position for health is well-documented (1–3). However, disease and mortality may not only be caused by the material conditions *per se* but can also be determined by status level (9). Psychosocial effects, such as stress related to relative deprivation and income inequality, can thus have an independent effect. Though associations between income inequality and health outcomes have been observed in several studies, the effects of inequality on health outcomes have been questioned and the possible pathways have not been definitely identified. A fundamental question is also at what geographical level we can expect this effect? In cross-country analyses, for instance, Wilkinson and Pickett (7, 8) have found associations between income inequality and poor health, but the effects at regional or local levels have been less clear.

With the Wilkinson and Pickett hypothesis as a point of departure, we herein examine the association between overall mortality and income inequality in the municipality of residence for Swedish residents aged 65–74 years. Though the inequality debate has been lively and population ageing is indeed one of today's key issues, the impact of income differentials on health outcomes in old age has not been a main focus in contemporary research. Under scrutiny here are those in an age group who have quite recently retired from working life and are increasingly facing health problems and higher mortality risks. The elderly are of special interest as their well-being and health rely on a well-functioning social security system.

The Swedish experience represents a comparatively equal society that has already passed several stages in the ageing transition. Nevertheless, the proportion of those aged 65 and older is expected to continue to increase from 18.2 per thousand in 2010 to 24.6 in 2050 (10). Life expectancy has continued to increase, now mainly through improvements in survival of the elderly. In 2011, in Sweden, females and males had a life expectancy of 83.7 and 79.8 years, respectively.

For our empirical investigations, we use a rich micro dataset to estimate the association between income inequality in Swedish municipalities and individual risk of death. Furthermore, we control for the impact of various confounders on individual and aggregate levels. More specifically, we examine how residing in municipalities with unequal vs. equal income distribution (using the Gini coefficient) is associated with the mortality of elderly people in Sweden in 2006, when controlled for the effects of absolute income, and average income level, as well as individual socioeconomic conditions (i.e. income, education, and family circumstances).

## Theoretical framework and previous studies

Differences in socioeconomic conditions are usually regarded as the main determinants of health and mortality disparities. Link and Phelan (11) even argue that social class is a ‘fundamental cause of death’ in itself; a person's position in the social hierarchy rather than the specific characteristics connected to this position is the main cause of class-specific mortality rates. One important point of departure for the lively debate on inequality and health emanates from the writings of Wilkinson and Pickett (7, 8). Claiming that income inequality adds to increasing health differentials in high-income countries, they base their argument on empirical observations of statistical associations between poor health outcomes and living in unequal societies. Based on these findings, they even maintain that the rich would gain from living in more equal societies.

The literature presents a variety of explanations for the expected impact of inequality on health outcomes. Subramanian and Kawachi (12) identify three main pathways of income inequality and health. First, the structural pathway implies that economic inequality leads to residential segregation which, in turn, results in negative health consequences. The second is the policy pathway, where economic inequality operates through general social and health-related policies. The third pathway emphasizes the role of social cohesion, sometimes conceptualized as social capital (13). A society characterized by a large amount of social capital takes good care of its citizens. Social capital can also provide citizens with a sense of belonging. In this way, psychosocial factors are introduced into the scientific debate; these factors have received increased attention in recent years, for example, in studies by Marmot (9) on the status syndrome. In line with this argument, Wilkinson and Pickett state that psychosocial conditions strongly affect the relationship between economic inequality and health outcomes.

Alternatively, Rodgers (14) has pointed to the so-called concavity effect and demonstrated that the marginal effect on improved health is stronger for the least wealthy. Thus, by raising the economic conditions of the poor, the general population's health would gain the most. This curvilinear association between income and health has been demonstrated in several studies (15, 16). A key question for further research is to investigate if and, in that case, to what extent the effect of inequality is larger on the lower income strata.

Obviously, the economic situation and social position of the elderly are strongly determined by previous income and occupation. Therefore, one may argue that conditions in the current residential region do not provide a full picture of the impact of inequality on health outcomes. However, we know that most elderly people are rather immobile and have lived in the same municipality for a long time, in many cases their entire adult life course (17). Even a cross-sectional analysis may give a good indication of how income inequality influences health outcomes.

Thus far, previous research provides an ambiguous picture (7, 12, 18–20). A recent cross-national study by Karlsson et al. (21), based on individual-level data from 21 countries, reports a negative relationship between income inequality and health in high-income countries while this was not the case in middle- and low-income countries. In a meta-analysis, Kondo et al. (22) found a modest adverse effect of income inequality on self-rated health and mortality. The association seems to be stronger in America than in Europe and on higher geographical levels (19).

A couple of previous studies in Sweden have not been able to verify any effect of income inequality on health and mortality. Gerdtham and Johansson (23) found, as expected, a clear effect of absolute income on all causes of mortality in Swedish municipalities but failed to find any effect of either mean community income (relative income) or income inequality. In a multilevel analysis, Henriksson et al. (24) found an association between income distribution and mortality in the 40–64 age group in Swedish municipalities when analyzed at the individual level, but this disappears when a multilevel model is applied.

## Study design, data, and methods

Identifying possible effects of income inequality on health outcomes require high-quality data with sufficient statistical power. Using multilevel methods, the data can be analyzed with individuals nested in their geographical locations (12, 19). While many previous studies have been based on small populations or aggregated data we have, for our purpose, access to a rich anonymized micro dataset, the so-called Linnaeus Database (25), containing, for instance, information from various administrative registers provided by Statistics Sweden as well as by the Swedish National Board of Health and Welfare. The data include the individual records of all residents in Sweden and contain rich information on socioeconomic conditions for each individual as well as their place of residence. From this dataset, we can aggregate data to any spatial unit and characterize any such unit by, for instance, level of income or education.

In this article, we analyze all-cause mortality among the elderly, aged 65–74 years, in 2006 on a municipality level. We identified every person in Sweden born between 1932 and 1941 who was alive on the last day of December 2005. The total sum of individuals was 793,380. The outcome variable was deaths during 2006, amounting to 12,326 cases. For each individual, we have information on municipality of residence, sex, marital status, and level of education on the last day of December 2005 and, finally, disposable income, individualized from family income for 2005. The disposable income variable is based on the total yearly family income from different sources such as income from work and self-owned firms, retirement pensions, social benefits, income from capital, and so on, minus tax and some other expenditure. This family income is divided by the number of family members and weighed by the age of the family members (26). Birth year controls for the age of the individuals. Marital status is categorized as unmarried, married (not including co-habitants), widowed, or divorced. Since almost all in the sample have left the workforce, occupational information is not included and, hence, level of education is used as an indicator of socioeconomic position. The different levels are primary education, lower secondary education, upper secondary education, tertiary education, and second stage of tertiary education. The income of the studied population is mainly based on their retirement pensions, something that should reasonably well reflect income positions during their working-active ages. We consider disposable income to be a good measure of their economic situation. The variable is categorized into five groups, from lowest to highest, of equal size. The cutoff points in SEK for each quantile are 90,200; 119,700; 152,500; and 207,100. The highest income is 78,191,700 SEK. Mean disposable income is 165,941 SEK, corresponding to 25,167 USD.

Apart from the individual variables, we construct variables describing the different residential contexts; in this case, the 290 Swedish municipalities for 2006, with a median population size of 15,252, maximum of 771,038 (Stockholm), and minimum of 2,553 (Bjurholm). The measures included are those on inequality in disposable income and mean disposable income in each municipality. Since we have information on income for each individual, we measure the level of income distribution using the Gini index. The Gini index is a commonly used measure of inequality, and portrays the share of income cumulatively earned from those with the lowest income up to the highest. In a society with complete income equality, the Gini coefficient is zero. If one person earns all the income (i.e. total inequality), the coefficient is one. The index is calculated based on a population aged between 25 and 60 years. As this is used as a characteristic of each municipality, we prefer to calculate it for an age group that is active in the workforce and not for the age group on which we are focusing in this study. We have used individualized disposable family income (for definition of disposable income, see above). For every municipality, we also calculate mean disposable income using the same age group as when calculating the Gini coefficient. Mean disposable income is a measure of the general economic standard in each municipality, thus allowing us to separate the association between health and income inequality from that between health and economic level.

In a first step, we explore the geographical patterns of income inequality, mean income and mortality. We thereafter analyze the question using aggregate data, with the outcome of the mortality rate of the municipalities for the 65–74 age group and its relation to inequality levels and mean disposable income. This is how many of the studies on the impact of inequality have been performed. A drawback to this approach is that we do not control for personal income levels. However, the main approach in this article is to utilize multilevel analysis with individual data and aggregate data hierarchically. We can, thus, compare results from different methods.

In a first micro model, the impact of the individual characteristics (individual income, age, sex, marital status and level of education) is investigated. Mean disposable income is introduced in the second model, and income inequality as measured by the Gini coefficient in the residential areas is investigated in the third. Finally, we also check for possible interaction effects between Gini and mean income in a fourth model in order to detect differences related to income levels.

The outcome variable is an indicator of the individual's death or survival. The traditional starting point for analyzing this type of dichotomous outcome variable is logistic regression, which is used in Model 1. In this model the link function describing the linear part of the model with the probability that the event occurs, in this case death, can be written as: $\text{log}(\frac{\pi_{i}}{1-\pi_{i}})=\beta \prime x_{i}$ where *π*_*i*_ denotes the probability of death for individual *i* and *β′* denotes the parameter row vector associated with the explanatory variables in vector *x*_*i*_ which starts with an intercept. It can be suspected that the risk of dying is associated with where they live. To take this into account, we extend the model with the term $u_{j}\sim N(0,\sigma_{j}^{2})$ capturing the municipality variation leading to the link function $\text{log}(\frac{\pi_{\mathrm{ij}}}{1-\pi_{\mathrm{ij}}})=\beta \prime x_{\mathrm{ij}}+u_{j}$. This two-level model is used in Models 2 to 4.

All statistical analyses as well as figures are produced within the R environment (27).

## Results

### Geographical patterns of economic conditions and mortality

**Fig. 1:**
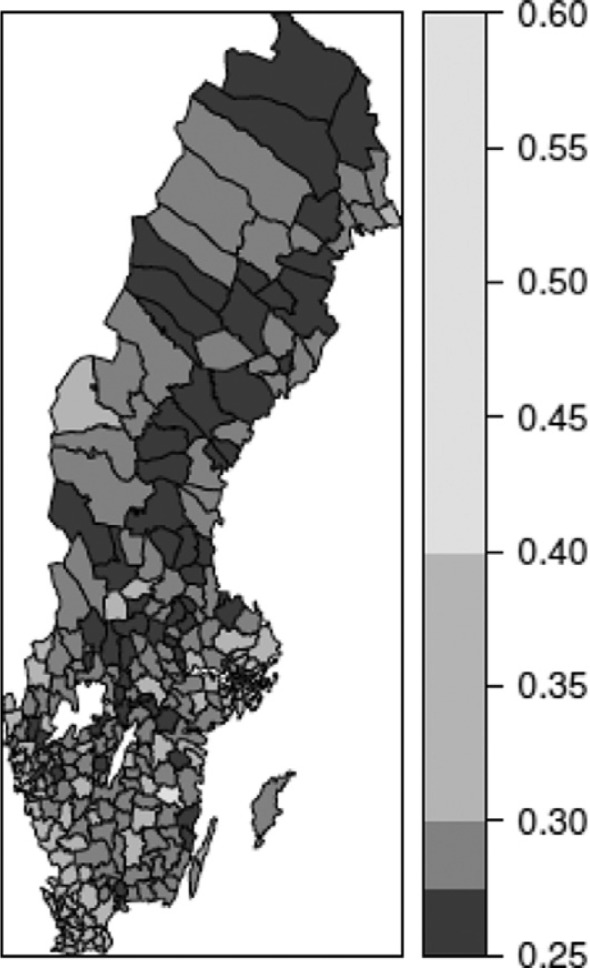
Income inequality using Gini coefficients in Swedish municipalities, 2006.

**Fig. 2:**
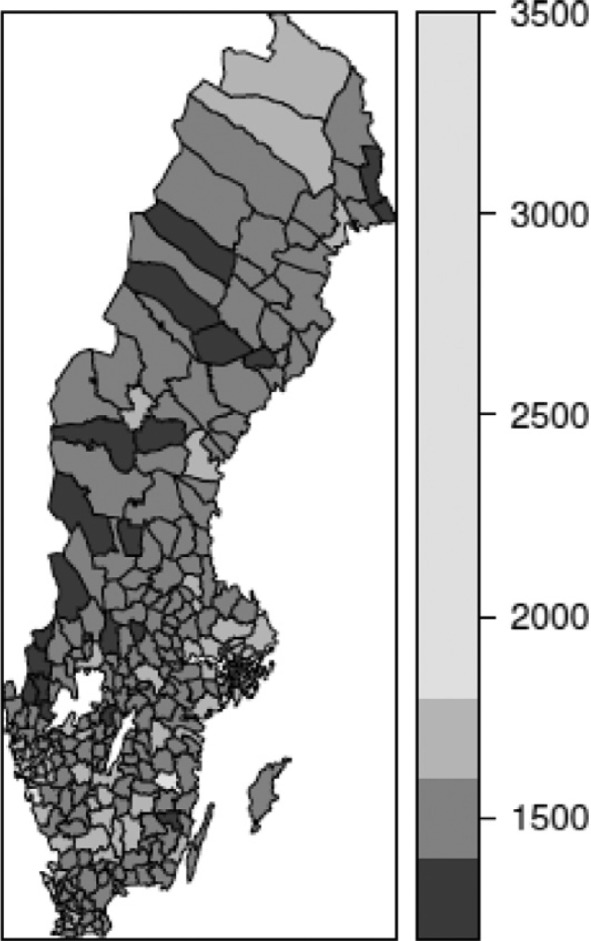
Mean disposable yearly income (in 1,000 SEK) in Swedish municipalities, 2006.

Mapping the geographical pattern of mean incomes and Gini on a municipal level (Figs. 1 and 2) reveals the variation in income distribution and some regional variations, whereby the most densely populated areas are characterized by higher levels of income inequality and higher mean incomes while the opposite is found in northern Sweden (white areas represent the largest lakes).

The visual impression of the figures is that of a strong connection between income inequality and mean income. This is confirmed by the correlation between the two variables reaching 0.734 (Pearson correlation), suggesting that wealthier municipalities have higher levels of inequality.

However, large income inequalities within municipalities do not necessarily indicate widespread poverty. Those with the lowest income in rich municipalities may very well be better off than those from other parts of the country. Nonetheless, within these wealthy places some have extremely high incomes, resulting in high levels of income inequality.

**Fig. 3:**
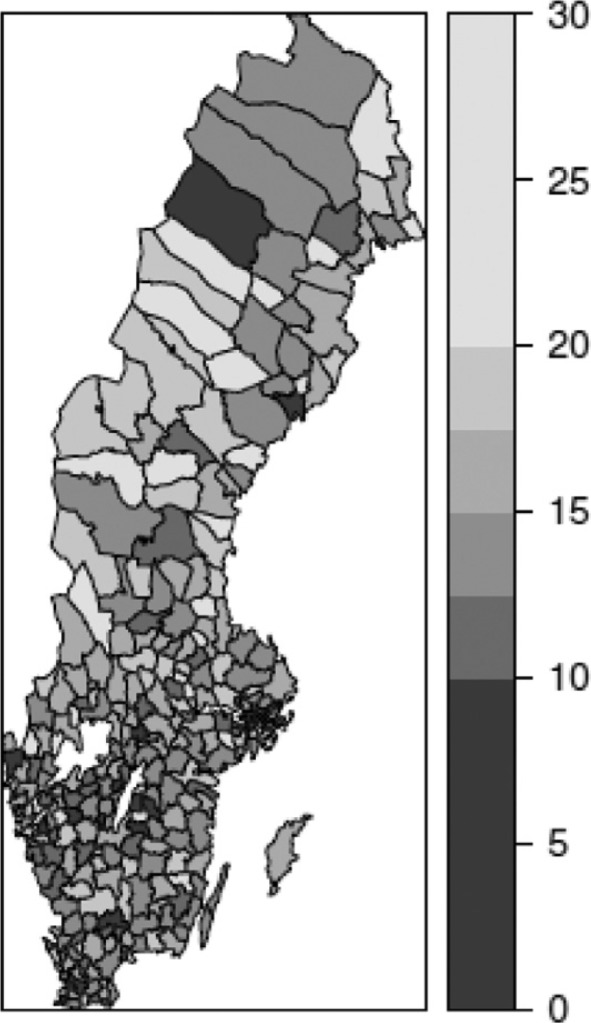
Mortality rate (deaths/1,000) in the 65–74 age group in Swedish municipalities, 2006.

Finally, the distribution of the mortality rate in the 65–74 age group in municipalities is presented in Fig. 3. The correlation between mortality in ages 65–74 years and the ecological variables is also significant, although less so than for the previous one. The correlation between mean income and mortality is −0.311, and between the Gini index and mortality is −0.222. In this case, random fluctuation has an impact on the aggregate figures, which also influence the correlation level.

### Model based on aggregate numbers

Table 1 presents the results from a model, including both mean disposable income and income inequality. According to this analysis, the mortality levels in municipalities are determined primarily by income level, while the inequality measure has no significant effect whatsoever in either direction. An analysis of aggregate data provides no support for the hypothesis that income inequality leads to higher mortality. This is in accordance with many other aggregate studies from different countries in which any effect on subnational levels has not been verified.

**Table 1:** Linear regression

	Parameter estimate	*P* value
Constant	0.025	0.000
Mean disposable income	−0.006	0.000
Income inequality, Gini coefficient	0.015	0.866

Mortality rate for the 65–74 age group dependent on mean disposable income and the Gini coefficient in Swedish municipalities, 2006.

### Models with individual data, nested in municipalities

In the next step, the association between income inequality and mortality were analyzed on the individual level, controlling for any impact from individual variables such as disposable income and level of education, as well as aggregated variables, including mean income in the municipalities.

The first analysis, including only data on an individual level (Table 2), revealed expected effects on mortality from income and levels of education, with a clear gradient from the poorest to the richest. Evidently, the mortality risks increased with age and were significantly lower for women. Similarly, as expected, being married or living in a partnership turned out to be beneficial for health.

**Table 2:** Logistic regression

	Model 1		Model 2		Model 3		Model 4	
				
	Est	*P* value	Est	*P* value	Est	*P* value	Est	*P* value
*Sex* (ref. female)								
Male	0.64	0.00	0.64	0.00	0.64	0.00	0.64	0.00
*Birth year*	−0.09	0.00	−0.09	0.00	−0.09	0.00	−0.09	0.00
								
*Marital status* (ref. married)								
Widowed	0.34	0.00	0.34	0.00	0.34	0.00	0.34	0.00
Unmarried	0.56	0.00	0.56	0.00	0.56	0.00	0.56	0.00
Divorced	0.50	0.00	0.50	0.00	0.50	0.00	0.50	0.00
								
*Education* (ref. second stage, tertiary educ.)								
No information	0.27	0.00	0.28	0.00	0.26	0.00	0.27	0.00
Primary education	0.14	0.00	0.14	0.00	0.14	0.00	0.14	0.00
Lower second. educ.	0.12	0.00	0.12	0.00	0.12	0.00	0.12	0.00
Upper second. Educ.	−0.21	0.00	−0.21	0.00	−0.21	0.00	−0.21	0.00
Tertiary educ.	−0.38	0.00	−0.38	0.00	−0.39	0.00	−0.38	0.00
								
*Disposable individual income*. (ref. mean)								
Highest	−0.34	0.00	−0.34	0.00	−0.34	0.00	−0.02	0.92
High	−0.18	0.00	−0.17	0.00	−0.17	0.00	−0.50	0.02
Low	0.20	0.00	0.20	0.00	0.20	0.00	0.37	0.08
Lowest	0.05	0.09	0.05	0.09	0.05	0.09	0.19	0.41
*Mean disposable income in municip*.			−0.05	0.00	−0.22	0.00	−0.21	0.01
*Income inequality in municip. (Gini)*					1.08	0.00	1.24	0.02
								
*Interaction disposable individual income/income inequality*								
Highest/Gini							1.05	0.13
High/Gini							−0.99	0.18
Low/Gini							−0.52	0.43
Lowest/Gini							−0.46	0.54
Intercept	165.04	0.00	165.24	0.00	165.07	0.00	164.90	0.00
AIC	1,23,519		1,23,513		1,23,509		1,23,508	

Deaths between the ages of 65 and 74 years in Sweden, 2006.

In a second model, we introduced mean income in the municipality in a multilevel analysis. We found a positive association of income level on the chance of surviving for both the rich and the poor and, although this new variable was introduced, almost no change in the other variables occurred.

The results from the third model confirm the income inequality hypothesis. A higher Gini coefficient is associated with a significantly higher rate of mortality. The effect of the other variables remains largely unchanged. Similarly, when controlling for possible interaction between individual income and income inequality in the last model, no interaction effect was found. In addition, since separate analyses of different income groups do not show any economic gradient in the inequality effects, we have not been able to confirm that large economic differences exposed poorer groups to a higher degree (results not shown). We have calculated VPC (Variance Partition Coefficient) according to the latent variable method. The VPC is the same for Models 2, 3, and 4, and is very small (0.0002968387).

### Expected death risks for hypothetical individuals

Compared to most other studies, this investigation is based on a much larger population. A consequence is that we easily get significant results that in practice have a very marginal impact on mortality risks. To evaluate the possible impact, we have calculated the effects measured in death risks depending on different personal as well as municipality characteristics (see Table 3). The variables personal disposable income, mean disposable income in municipality, education, and birth year are fixed, while sex, marital status, and Gini coefficient can vary. The death risks increase from 9/1,000 for females to 16.9/1,000 for males, a substantial increase. Being an unmarried male compared to a married male increased the risk even more. An increase in Gini coefficient in the municipality did not have an equally strong effect. Yet, it represents an increase from 16.9/1,000 to 18.6/1,000, as a comparison between lines two and four shows. This is not a negligible difference, as it corresponds to more than a fourth of the well-established male/female difference.

**Table 3:** Estimated expected risks of death for four hypothetical individuals in Sweden, 2006

Sex	Marital status	Disp/1,000	Education	MeanDisp/1,000	Gini	Birth year	Death risk
Female	Married	1.63	Upper sec.	1.63	0.3	1936	0.00899
Male	Married	1.63	Upper sec.	1.63	0.3	1936	0.01685
Male	Single	1.63	Upper sec.	1.63	0.3	1936	0.02916
Male	Married	1.63	Upper sec.	1.63	0.4	1936	0.01858

## Discussion

Since socioeconomic conditions are identified as key issues for public health in both high- and low-income countries, the lively debated question introduced by Wilkinson (7, 8), as to how inequality influences health outcomes, is certainly an issue of concern from a global perspective as well. Similarly, with increasing life expectancy, the impact of income inequality on health conditions among the elderly is of great concern in countries with different levels of development. With this background, and due to rather ambiguous results from previous studies, here we have contributed to the research on health outcomes and income inequality in old age using a unique micro dataset on Swedish municipalities.

Previous research supporting the Wilkinson hypothesis is mainly based on cross-national analyses or on data from larger regions such as American states. In contrast, most studies on small units such as neighborhoods, parishes, or local communities have not supported the hypothesis (12, 19). Wilkinson and Pickett (8) maintain that the weaker association found at a lower level of aggregation is an expected outcome, since social forces at higher levels shape spatial income distribution and may hide the impact of inequality on health outcomes. However, with our high-quality data, we find contrasting results.

In the first step, we used aggregate data to test the hypothesis and, in line with previous studies, found no association between income inequality and mortality rates in the analysis on an aggregate level. In the analysis on an individual level we found, as expected, clear associations between risk of death and mean disposable income in the municipalities. In addition, we found that mean income has a strong effect in itself, regardless of personal income. Both rich and poor seem to gain from living in a place with a high income level.

However, our main contribution stems from our further micro analysis, showing a significant association between income inequality in Swedish municipalities and individual risk of death among people aged 65–74 years. This association contrasts fundamentally with that of the aggregate level analysis, a common but partly inadequate method. Our results, based on a more accurate method, illustrate the importance of using individual data.

In international comparisons by Wilkinson and Pickett (7, 8), Sweden represents a country with low levels of inequality and high life expectancy, thus representing a positive verification of their hypothesis. Sweden as well as the other Nordic countries have among the lowest national Gini coefficients – around 0.25 – in the world in comparison with, for example, the United States with a level of about 0.40, and the most unequal societies such as Brazil, with a level of around 0.60 (28). However, our results show that even in a comparatively equal society the level of income inequality needs to be considered.

Our results differ from Henriksson et al. (24) who found that, when analyzed properly in a multilevel analysis, the effect of income inequality disappeared and from the study by Gerdtham and Johansson (23) who only found an effect of absolute income. The explanation for the different results requires further investigation; however, one important aspect which may have been crucial for this is that Henriksson et al. restrict their analysis to 40–64 year olds, that is, an age in which mortality is lower than among the elderly. The lower number of fatal cases may also have an impact on the results. Obviously, we must take into consideration diverging patterns in different age groups. A study by Dahl et al. (29) on mortality in Norway has reached similar results as in the present study; they found a significant (but partly modest) effect of income inequality. They do however investigate this on a higher geographical level than municipalities – the economic regions – and argue that we would expect less or no effects at smaller area size.

Some circumstances, however, could make it more plausible that income inequality is more important in working age. Swedish public pensions compress the income distribution, making the inequalities smaller than in younger age groups (30). Furthermore, some researchers argue that differences become smaller due to selection effects and/or the stronger impact of biology in old age (30–33). In our case, we find a clear association between income and mortality in our group of elderly. Even if we have not compared directly with conditions in other age groups, the results may support the cumulative advantage hypothesis (5). According to this hypothesis, differences related to socio-economic status increase throughout life and, thus, become even more apparent in old age. Thus, the effect of socio-economic conditions may differ between those still in the workforce and those who have retired and, moreover, between younger and older pensioners.

We would like to emphasize two important aspects of our findings. The first is that we find effects of income inequality in one of the most equal countries in the world. So far, the evidence has primarily been taken from societies with much more unequal income distributions. Here, the findings show that this effect can be observed even in a society with strong income redistribution and institutions that should take care of many of the disadvantages of belonging to a group with fewer economic resources. Our results are thus in line with those of Dahl et al. (29), although at a different geographical level. The other noteworthy result, which contradicts many previous assumptions and earlier studies, is that the effect is also established on local levels, something that has been questioned with the assumption that there are other processes of segregation taking place in local places and that these processes would make it impossible to identify any effect. We believe that these findings force us to reconsider the issue, constituting a challenge to develop study designs that can disentangle the problem.

### Study limitations and future research

Although we find an association between level of income inequality and mortality, the exact pathway is not known. Several alternatives have been discussed in the literature (12, 20). The psychosocial effect described as relative deprivation, suggested by Wilkinson and Pickett (8), is only an alternative. Another is the observation, made by Rodgers (14) and further discussed by Fritzell (16, 34), that the marginal effect on health is stronger for the poorest groups. Consequently, societies where conditions for those with the lowest incomes are improved and, thus, income inequality is decreased will present better health results.

Another possible pathway refers to the effects of local municipality politics, that is, a neo-material explanation (19). A study by Rostila et al. (35) suggests that reduced spending on social goods could explain the association between income inequality and self-rated health at municipality level in the county of Stockholm. Swedish municipalities tax their citizens and have powers of decision in many matters related to health care and care of the elderly. Different municipalities may spend differently on infrastructure. An unequal municipality may be less interested in allocating resources to the most needy; on the other hand, they may gain from a generally good infrastructure and good quality of care for the elderly. However, based on these preliminary findings, we must admit that we cannot distinguish the effects of the possible pathways. We need to develop a design for analyzing this in our future studies.

Our plan for the future is to continue investigating at different geographical levels. A reasonable assumption is that the effect would be stronger within local labor market regions, since they are more integrated functional regions and social comparisons are more easily made on this level. We will also introduce a longitudinal perspective and analyze the effects from a life course perspective, following the individuals from working age until old age. In this way, we can control for the possible effects of migration and segregation caused by people moving. Subramanian and Kawachi (12) suggest that income inequality has its strongest effect up to 15 years later. In the near future, we will be able to follow individuals continuously from 1986 to 2009 and also add economic, family, and residential information from the 1960, 1970, and 1980 censuses.

Finally, our study indicates the need to analyze and compare the effects on different age groups. Is there a cumulative (dis-)advantage effect throughout the life course? (5). The comparison with other studies might be an effect of different patterns depending on the age studied. People of working age may have a different pattern. We would also like to compare the effects we have found with that on the oldest age groups when disease and death become even more apparent.

## Conclusion

This study provides us with another piece in the understanding of the role of income inequality for health and mortality. The results give a partly different picture from that of many other studies. We have found that income inequality is a factor to consider even in a comparatively equal country as Sweden and that it has an impact also in local environments. In our case, we have been able to identify this association among the elderly. However, the exact pathways are still unclear, but we hope to bring more clarity on this in our future studies.

Finally, even if most studies on the association between inequality and poor health have focused on the wealthiest nations, it has, and will even more so in the future have, a strong global relevance. For the many transitional countries in Latin America, Asia, and Africa, the consequences of inequality on the health outcomes of the ageing population should be an issue of great concern.
